# Supplementary material for: SCFFBXW11 Complex Targets Interleukin-17 Receptor A for Ubiquitin–Proteasome-Mediated Degradation
Source: Biomedicines. 2024 Mar 28;12(4):755. doi: 10.3390/biomedicines12040755 (PMC11047997; doi:10.3390/biomedicines12040755)
Supplement: Supplementary file 1 [file biomedicines-12-00755-s001.zip › biomedicines-2915539-supplementary.pdf]

Supplementary figures

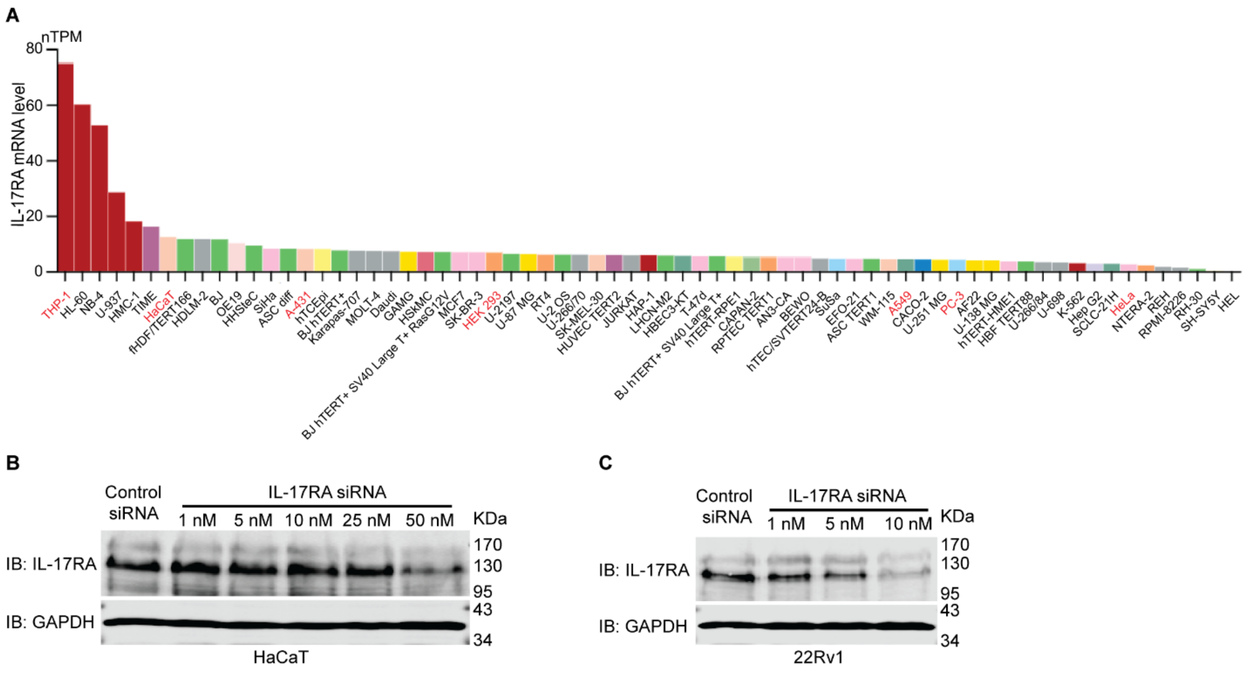

Figure S1. Expression level of endogenous IL-17RA mRNAs across multiple cell lines and validation of the specificity of anti-IL-17RA antibody. (A) Human Protein Atlas database (https://www.proteinatlas.org/; access date 10/27/2022) was explored to figure out mRNA levels of IL-17RA across multiple human cell lines. **(B & C)** To validate the specificity of anti-IL-17RA antibody (G9 clone, Santa Cruz Biotechnology), HaCaT cells in 10-cm dishes (B) and 22Rv1 cells in 6-cm dishes **(C)** were transiently transfected with various amounts of IL-17RA siRNA or control siRNA for 48 h. Protein levels of endogenous IL-17RA were analyzed using Western blot.

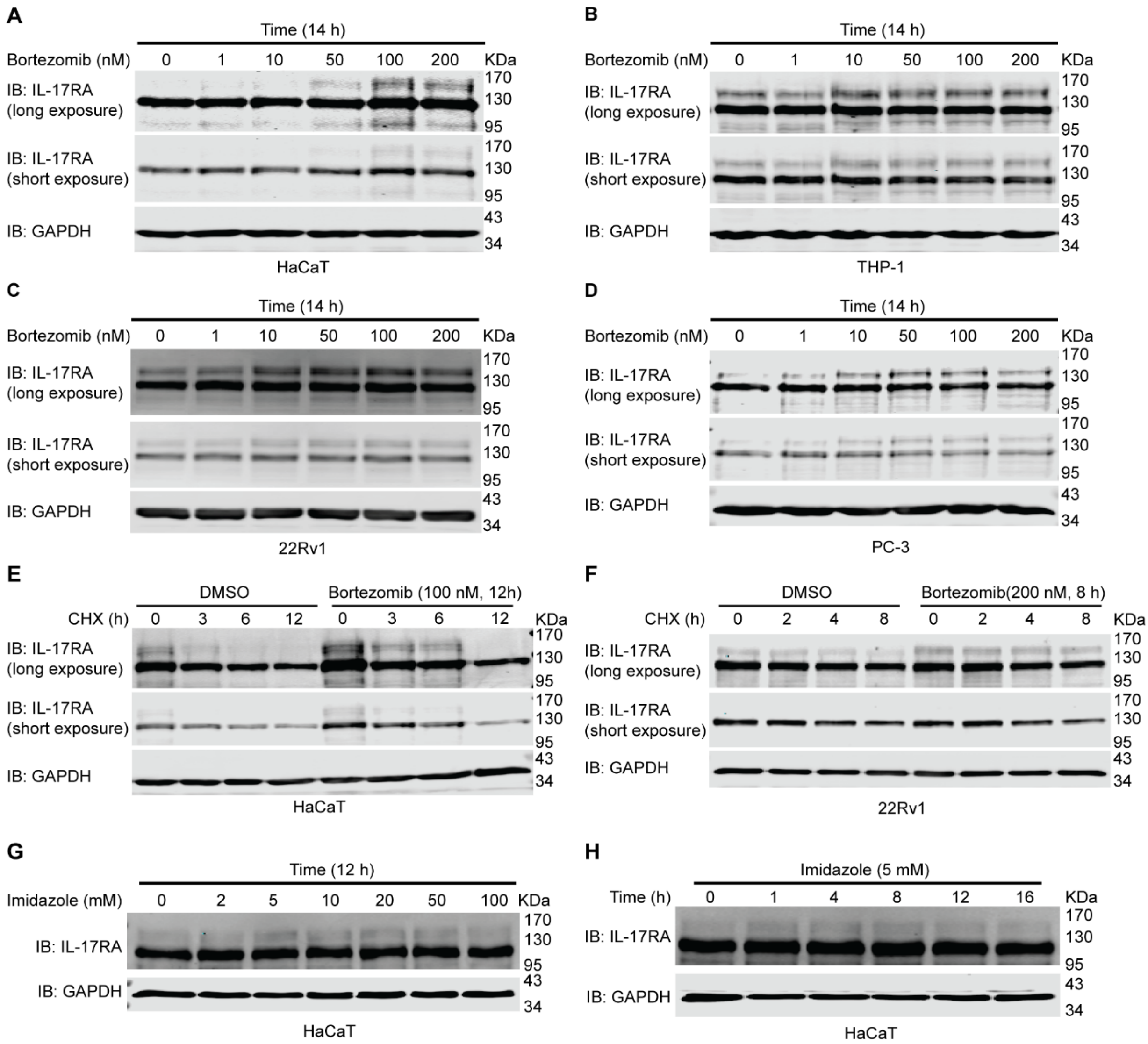

Figure S2. Proteasome inhibitor bortezomib slightly accumulates endogenous IL-17RA but lysosome inhibitor imidazole has no obvious effect on IL-17RA protein levels. (A-D) Western blot analysis of endogenous IL-17RA in HaCaT (A), THP-1 (B), 22Rv1 (C), and PC-3 (D) cell lines treated with 1 nM, 10 nM, 50 nM, 100 nM, and 200 nM bortezomib for 14 h. DMSO was used as control treatment. **(E)** Western blot analysis of endogenous IL-17RA in HaCaT cell line treated with 100 nM bortezomib for 12 h and 50 μg/ml CHX for indicated time. DMSO was used as control treatment. **(F)** Western blot analysis of endogenous IL-17RA in 22Rv1 cell line treated with 200 nM bortezomib for 8 h and 50 μg/ml CHX for indicated time. DMSO was used as control treatment. **(G)** Western blot analysis of endogenous IL-17RA in HaCaT cell line treated with 2 mM, 5 mM, 10 mM, 20 mM, 50 mM, and 100 mM imidazole for 12 h. DMSO was used as control treatment. **(H)** Western blot analysis of endogenous IL-17RA in HaCaT cell line treated with 5 mM imidazole for indicated time. DMSO was used as control treatment. Treatment of bortezomib was repeated 3 times while treatment of imidazole was repeated one time.

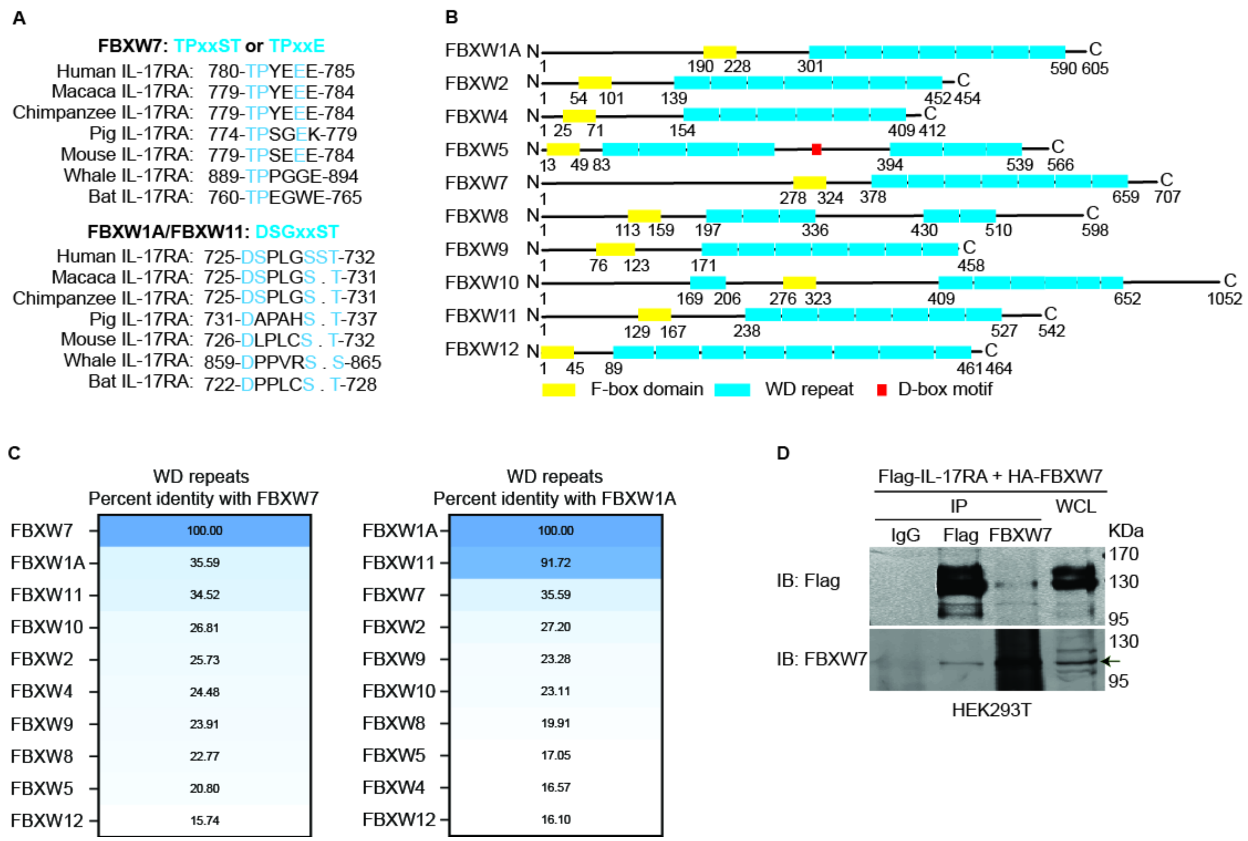

Figure S3. F-box and WD repeat domain containing 7 (FBXW7) and FBXW1A/11 are E3 ligase candidates recognizing IL-17RA phosphodegron. (A) Phosphodegron TPxxE recognized by FBXW7 matches amino acid 780-785 of human IL-17RA. Phosphodegron DSGxxST recognized by FBXW1A/11 matches amino acid 725-732 of human IL-17RA. The phosphodegrons are conserved across different species. **(B)** Diagram showing conserved domains, F-box domain and WD repeat domain, of FBXW family members. FBXW5 has a special D-box domain. **(C)** Percent identity of tryptophan-aspartic acid (WD) repeat domains of FBXW family members was computed with Clustal Omega algorithm [75]. The plot was made using Prism GraphPad. **(D)** Binding of IL-17RA with FBXW7. HEK293T cells were seeded into 6-cm dishes at the density of 1 x 10^6. 1.5 μg full-length Flag-IL-17RA and 1.5 μg full-length HA-FBXW7 plasmids were transiently transfected using jetPRIME transfection reagent. An empty vector was used to compensate for the total amount of plasmids. 48 h post transfection, proteins were extracted using IP lysis buffer. The co-IP assays were carried out using 2 μg normal IgG (Cell signaling technology, #2729), 2 μg anti-Flag M2 or 12 μg anti-FBXW7. Experiments were repeated 4 times independently. Arrow indicates the band of FBXW7.

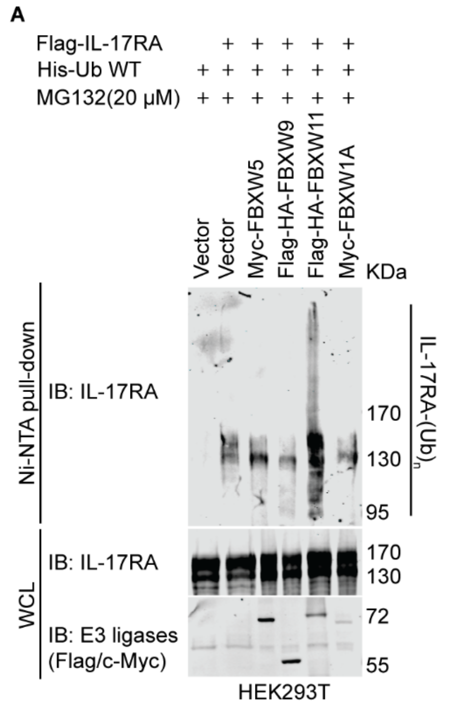

Figure S4. FBXW11 has the highest ubiquitylation activity towards IL-17RA. (A) 21 h before transfection, HEK293T cells were seeded into 10-cm dishes at a density of 4.5 x 10^6. 1.5 µg full-length Flag-IL17RA, 3 µg His-ubiquitin WT, 2 µg Myc-FBXW5, 1.5 µg Flag-HA-FBXW9, 3.5 µg Flag-HA-FBXW11, 3.5 µg Myc-FBXW1A, and various amounts of empty vector (to compensate for the total amount of plasmids) were transiently transfected using jetPRIME transfection reagent as indicated; 40 h post transfection, 20 µM MG132 was added to treat cells for 8 h.

A

B

C

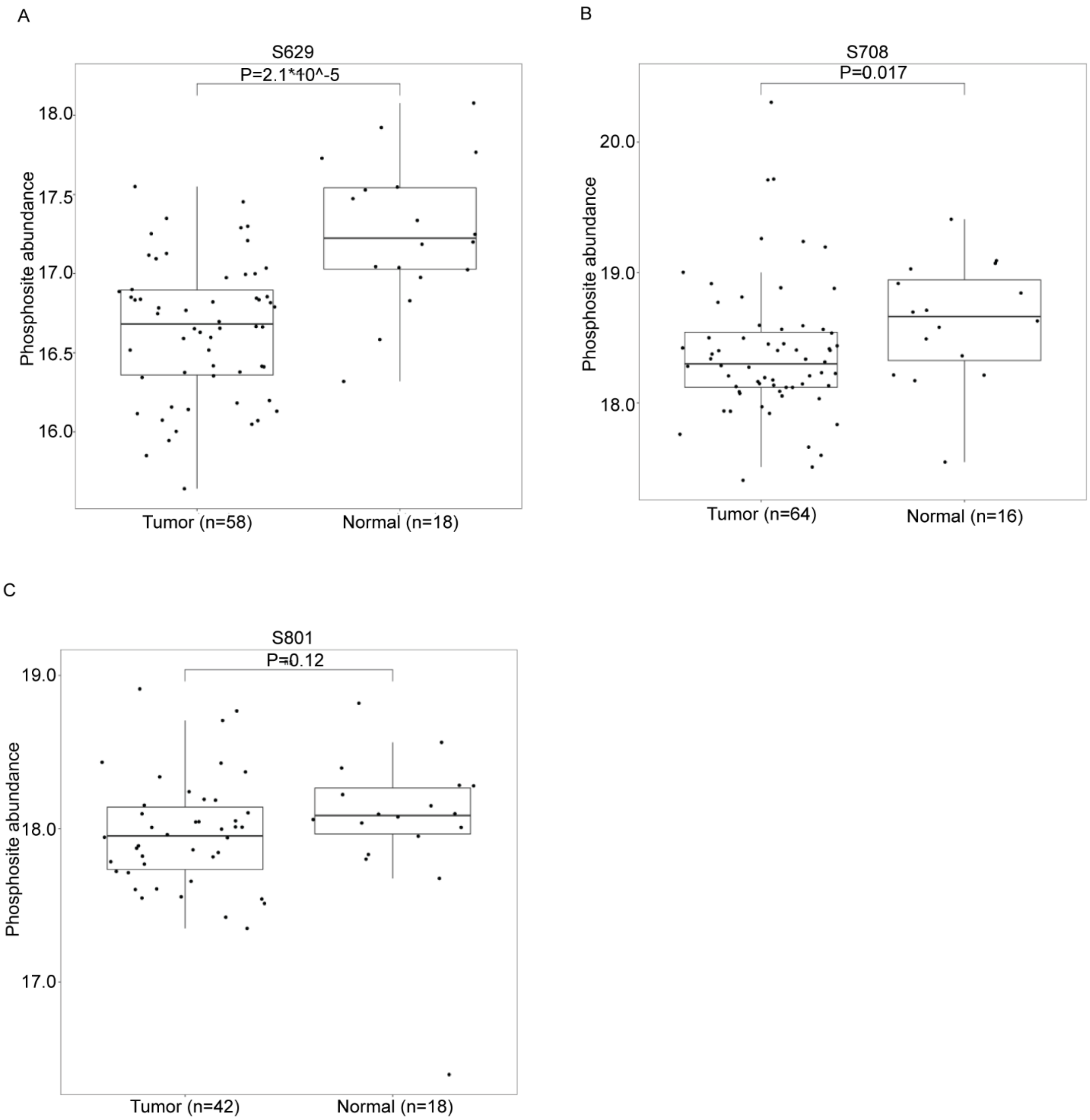

Figure S5. Phosphosite abundance of IL-17RA in UCEC and Normal tissue. (A) Phosphosite abundance data obtained from the LinkedOmicsKB platform showed that phosphorylation of S629 in UCEC was significantly lower than normal control. **(B)** Phosphosite abundance data obtained from the LinkedOmicsKB platform showed that phosphorylation of S708 in UCEC was significantly lower than normal control. **(C)** Phosphosite abundance data obtained from the LinkedOmicsKB platform showed that phosphorylation of S801 in UCEC was slightly lower than normal control without statistical significance.

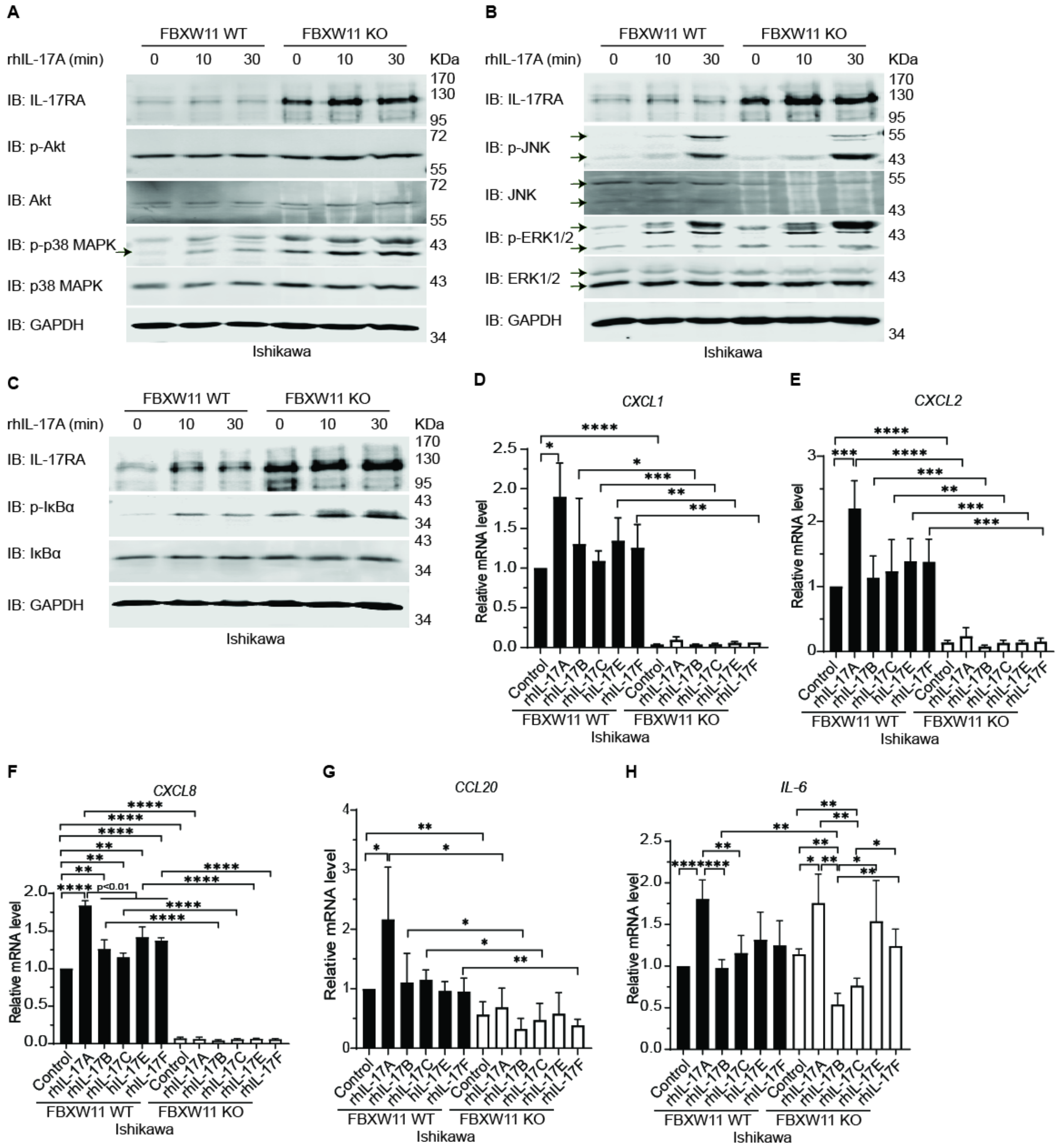

Figure S6. Knock-out of FBXW11 suppresses expression of IL-17-downstream genes. (A-C) Western blot analysis of IL-17RA, phosphorylated AKT (p-AKT), AKT, phosphorylated p38 MAPK (p-p38 MAPK), p38 MAPK, phosphorylated JNK (p-JNK), JNK, phosphorylated ERK1/2 (p-ERK1/2), phosphorylated IκBα (p-IκBα) and IκBα. 2 x 10^6 Ishikawa FBXW11 WT cells and 2.5 x 10^6 Ishikawa FBXW11 KO cells were treated with 20 ng/ml rhIL-17A for 10 min and 30 min, while the control cells were treated with 0.1% BSA. Experiments were repeated at least 5 times independently. Arrows indicate the band of p-p38 MAPK, p-JNK, JNK, p-ERK1/2, and ERK1/2. **(D-H)** Induction of IL-17-downstream gene expression. Ishikawa FBXW11 WT and FBXW11 KO cells were treated with 20 ng/ml recombinant human IL-17 (rhIL-17) cytokines, including rhIL-17A, rhIL-17B, rhIL-17C, rhIL-17E, and rhIL-17F, for 2 h. Expression of *CXCL1* (D), *CXCL2* (E), *CXCL8* (F), *CCL20* (G), and *IL-6* (H) was evaluated using real-time qPCR analysis, normalized to internal GAPDH control. The cells treated with 0.1% bovine serum albumin (BSA) were used as calibration control. Fold change of each target gene over control is shown. Error bar represents mean ± standard deviation (S.D.). The student's t test

was used to calculate statistical significance of fold change. * $P < 0.05$, ** $P < 0.01$, *** $P < 0.001$, and **** $P < 0.0001$. Experiments were repeated 3 times independently.
